# Are treatment effect assumptions in orthodontic studies overoptimistic?

**DOI:** 10.1093/ejo/cjab018

**Published:** 2021-05-15

**Authors:** Jadbinder Seehra, Daniel Stonehouse-Smith, Martyn T Cobourne, Michail Tsagris, Nikolaos Pandis

**Affiliations:** 1 Department of Orthodontics, Faculty of Dentistry, Oral & Craniofacial Sciences, King’s College London, Floor 25, Guy’s Hospital, Guy’s and St Thomas NHS Foundation Trust, London, UK; 2 Department of Economics, University of Crete, Rethimnon, Greece; 3 Department of Orthodontics and Dentofacial Orthopedics, Dental School/Medical Faculty, University of Bern, Bern, Switzerland

## Abstract

**Background:**

At the clinical trial design stage, assumptions regarding the treatment effects to be detected should be appropriate so that the required sample size can be calculated. There is evidence in the medical literature that sample size assumption can be overoptimistic. The aim of this study was to compare the distribution of the assumed effects versus that of the observed effects as a proxy for overoptimistic treatment effect assumptions at the study design stage.

**Materials and method:**

Systematic reviews (SRs) published between 1 January 2010 and 31 December 2019 containing at least one meta-analysis on continuous outcomes were identified electronically. SR and primary study level characteristics were extracted from the SRs and the individual trials. Details on the sample size calculation process and assumptions and the observed treatment effects were extracted.

**Results:**

Eighty-five SRs with meta-analysis containing 347 primary trials were included. The median number of SR authors was 5 (interquartile range: 4–7). At the primary study level, the majority were single centre (78.1%), utilized a parallel design (52%), and rated as an unclear/moderate level of risk of bias (34.3%). A sample size was described in only 31.7% (110/347) of studies. From this cohort of 110 studies, in only 37 studies was the assumed clinical difference that the study was designed to detect reported (37/110). The assumed treatment effect was recalculated for the remaining 73 studies (73/110). The one-sided exact signed rank test showed a significant difference between the assumed and observed treatment effects (*P* < 0.001) suggesting greater values for the assumed effect sizes.

**Conclusions:**

Careful consideration of the assumptions at the design stage of orthodontic studies are necessary in order to reduce the unreliability of clinical study results and research waste.

## Introduction

The aim of healthcare research is to ultimately benefit patients by providing effective and useful therapies. There is evidence that studies with significant results are more likely to be published and to be published more expediently (1). In the context of academic success and under the ‘publish or perish’ principle, researchers have strong incentives to publish their results quickly, even if this means conducting small studies with stretched out analyses that are unlikely to be reproducible (2). Sample size calculations are an important component of clinical trial design that require assumptions and assumed choices, which are prone to manipulation. For a continuous outcome, a clinically relevant effect size and variance must be selected, and assuming a large difference and/or small standard deviation will reduce the required sample size and vice versa. Sample sizes can therefore be manipulated by selecting a large but unrealistic difference to be detected and/or a small standard deviation. Such a trial, although seemingly with adequate power at the design stage to detect the assumed effect, will be less likely to detect a smaller but possibly clinically relevant effect. Assumptions on effect sizes should be guided by clinical relevance and where feasible, based on existing evidence or from piloting and not based on a sample size that can be gathered. In reality, common approaches may include but are not limited to the following: 1. empirical estimates from published studies or pilot studies, 2. a priori, usually arbitrary, statements of clinical significance, and 3. convenience samples. There is evidence in the medical literature that assumptions during sample size calculation can be overoptimistic (3–5) often applying the ‘samba calculation’ or the ‘delta’ inflation method where investigators can start with the number of available participants and adjust the required assumptions to justify their sample size (6). Such practices may cast doubts in the process and can result in research waste.

Dental research has in general, been characterized by small studies (7–9) often with no involvement of a methodologist or prior protocol registration (10) and with reporting of predominantly statistically significant results (11, 12). Reporting of a priori sample calculation is not universal and varies across study designs with sample size calculations more commonly observed in interventional studies (13). There is some evidence that multicentre trials can include a larger sample size compared to single-centre trials (14). A systematic assessment of those assumptions compared to the final effects has not been undertaken in the field of dentistry and orthodontics. Therefore, it is the aim of this study to compare the distribution of assumed effects versus that of the observed effects in orthodontic primary studies reported in systematic reviews (SRs) as a possible indicator for overoptimistic treatment effect assumptions at the trial design stage.

## Materials and methods

### Eligibility criteria

We included orthodontic SRs published over a 10-year period between 1 January 2010 and 31 December 2019. The justification for this approach was based on the assumption that these SRs would include the best and most clinically relevant studies. To be included, the SR should include at least one meta-analysis on continuous outcomes, report the required study characteristics, be published in English, and report interventional procedures in orthodontic clinical trials involving human participants. Where multiple versions of the same systematic review existed, the latest version was selected.

### Search and selection of SRs

An electronic database (Medline via PubMed) search was undertaken using the following search terms: ‘orthodontic’ AND ‘systematic review’ OR ‘meta-analysis’. All relevant orthodontic SRs published in the Cochrane Library were also screened. All titles and abstracts were initially screened by one author (JS). Full-text articles of abstracts meeting the inclusion criteria were retrieved and further analysed for eligibility. Any disagreements in the final SRs were resolved by discussion among two authors (JS and DSS).

### Data extraction

Data regarding the sample size calculation was extracted from the individual trials included in the SRs. Prior to full data collection, data from five SRs was extracted by three authors (JS, DSS, and NP) independently. This pre-piloting process was undertaken to ensure consistency between authors regarding the interpretation of both data variables and forest plots. Consequently, all study characteristics were extracted by a single author (DSS) and entered into a pre-piloted Microsoft Excel® (Microsoft, Redmond, WA) data collection sheet. A second author (JS) reviewed the collected data. Any disagreements were resolved by discussion. At the SR level, the following information was extracted: number of authors, continent of the corresponding author, year of publication, PROSPERO registration and type of review (Cochrane and non-Cochrane). The following information from the SRs at the primary study level was extracted where available: continent of the corresponding author, year of publication, type of study, research setting (single or multi-centre), risk-of-bias assessment, and description of sample size calculation (assumed effect size, whether the effect size was based on evidence, level of power and actual reported effect size). When more than one meta-analysis was present, the meta-analysis directly related to the main outcome of the study was selected. When two or more meta-analyses were related to the main outcome, the meta-analysis with the greatest number of primary studies included was selected. Only parallel studies were included and for studies where the assumed difference at the design level was provided, that treatment effect was used to compare with the observed effect. In the absence of the assumed effect at the design stage the assumed effect was recalculated using the formula below by solving for the assumed treatment effect δ:


$$\begin{array}{l} Power=\Phi (\sqrt{\eta }\;\delta -z_{1-\alpha /2})+\Phi (-\sqrt{\eta }\;\delta -z_{1-\alpha /2}) \end{array}$$

Φ(.) *is the cumulative density function of the standard normal distribution*

$\delta =(\mu_{\alpha }-\mu_{0)}/\sigma \;\;\;$ *is the effect size*

$z_{1-\alpha }\;is\;the\;\left(1-\alpha \right)$ *quantile of the standard normal distribution and a is the significance level*

In other words, the reported power, sample size, and variance in the study were used for a backward calculation of the assumed effect. The assumption was that the sample size chosen was based on an assumed effect, variance, and power level to be calculated. The formula was entered into R software to facilitate the calculation of the assumed treatment effect δ from the formula. To carry out the calculation of the assumed treatment effect δ the assumed power and alpha and reported sample size and variance were used.

### Statistical analysis

Descriptive statistics were calculated at the SR, meta-analysis, and at the individual study level. Fisher’s exact test was used to assess potential associations between studies where sample size could be recalculated from the provided data and the following characteristics: assumed clinical difference stated and assumed clinical difference evidence based. The distribution of the assumed and observed effect size was plotted and the one-sided signed rank test for paired observations was used to statistically compare the assumed and observed effect sizes. All analyses were conducted using Stata 16.1 (StataCorp, College Station, Texas, USA) and R Software version 3.6.1 (R Foundation for Statistical Computing, Vienna, Austria).

## Results

**Figure 1.**
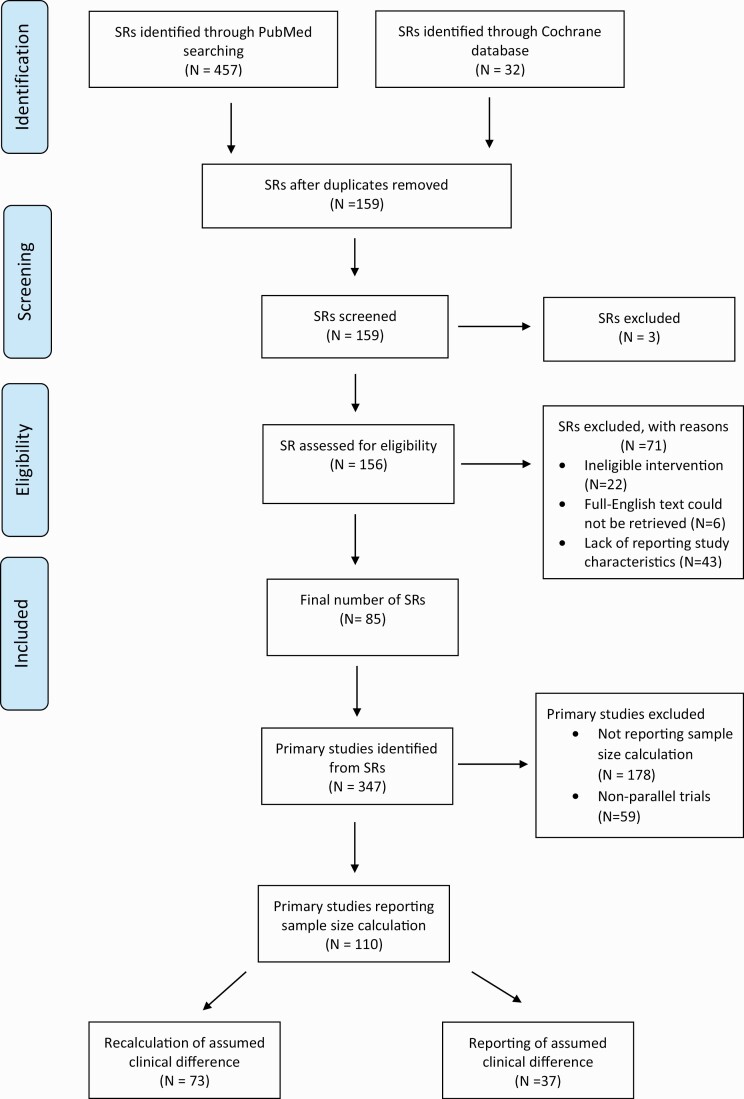
Flow diagram for systematic review and primary studies identification.

A total of 85 SRs with meta-analyses published between 2010–19 were included in the analysis (Figure 1). The SR study characteristics are shown in Table 1. Within the 85 SRs, 347 trials were identified (Figure 1). The median number of authors was 5 (interquartile range [IQR]: 4–7). At the primary study level, the majority were single centre (78.1%), utilizing a parallel design (52%), and rated as having an unclear/moderate level risk-of-bias (34.3%) (Table 2). In the initial cohort of 347, the sample size was described in only 31.7% (110/347) of studies. From the cohort of these 110 studies, only 37 reported the assumed clinical difference that the study was designed to detect; however, as explained earlier, the assumed treatment effect was calculated for the remaining 73 studies.

**Table 1.** Systematic review and meta-analysis characteristics. (SR dated 2020 was advance online publication and it was identified and accessible in the study search dates).

Systematic review characteristics	Median (IQR)	N (%)
Year of publication (N = 85)		
1) 2010		2 (2.4)
2) 2012		3 (3.5)
3) 2013		7 (8.2)
4) 2014		10 (11.8)
5) 2015		9 (10.6)
6) 2016		12 (14.1)
7) 2017		15 (17.6)
8) 2018		20 (23.5)
9) 2019		6 (7.1)
10) 2020		1 (1.2)
Number of authors (N = 85)	5 (4–7)	
Continent of corresponding author (N = 85)		
1) Europe		35 (41.2)
2) Americas		15 (17.6)
3) Asia or other		41 (41.2)
PROSPERO registration (N = 85)		
1) Yes		28 (32.9)
2) No		57 (67.1)
Type (N = 85)		
1) Cochrane		13 (15.3)
2) Non-Cochrane		72 (84.7)
Meta-analysis model (N = 66)		
1) Fixed		17 (25.8)
2) Random		49 (74.2)
Heterogeneity (I^2^) (N = 66)	60% (16%–85%)	
tau^2^ (N = 42)	0.35 (0.04–0.84)	

**Table 2:** Primary study characteristics.

Primary study characteristics	N (%)
Center (N = 288)	
1) Single	225 (78.1%)
2) Multi	27 (9.4%)
3) Practice	20 (6.9%)
4) Not reported	16 (5.6%)
Type of studies (N = 288)	
1) Parallel	150 (52.0%)
2) Split mouth	16 (5.6%)
3) Retrospective	55 (19.1%)
4) Prospective	67 (23.3%)
Risk of bias (N = 347)	
1) High	101 (29.1%)
2) Low	55 (15.9%)
3) Unclear/moderate	119 (34.3%)
4) Not undertaken	72 (20.7%)

**Figure 2.**
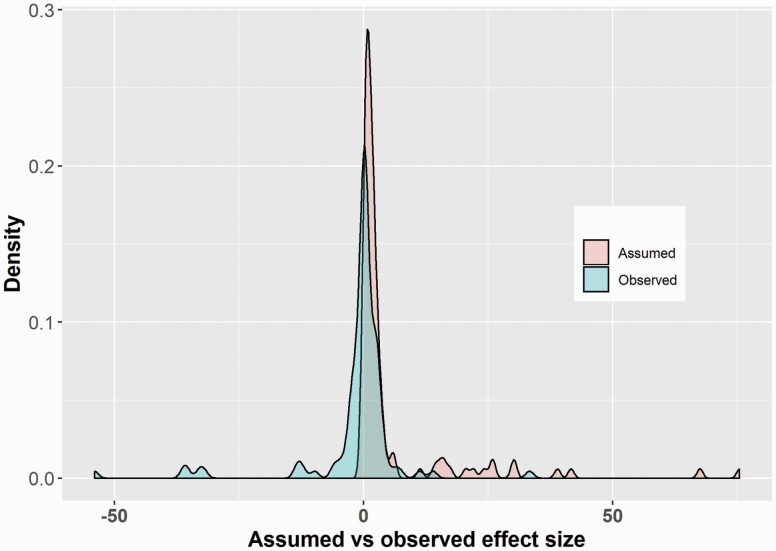
The difference between the assumed clinical difference and actual reported difference (N = 110).

The most frequently assumed power level was 80% (N = 53) and 90% (N = 34) with one study selecting a very low and unusual power level of 46%. The difference between the assumed clinical difference and actual observed/reported difference is shown in Figure 2. The one-sided exact signed rank test showed that the assumed effects are on average larger than the observed treatment effects (*P* < 0.001, 95% CI: 1.20, +inf).

## Discussion

The ethical merits of small clinical trials have been debated for a number of years (15, 16). Clinical researchers in orthodontics may face a dilemma between factors such as academic and clinical interests and trial feasibility when determining what is a meaningful effect size during their sample size calculation. By definition, small yet clinically significant effects require larger sample sizes (17). It can be argued that a priori assumed and expected treatment effects are based on clinical importance and relevance, but this may not corroborate with true treatment effects. For instance, a researcher would like to power a study to make sure that a certain effect size is not missed. Therefore, smaller treatment effects may not be of interest to be detected thus supporting the prior choices. In addition, if the true treatment effect had been known there would be no need to carry out the trial. Post hoc power calculations are not justified (18, 19), and the problem lies with the possibility of practices in which the assumed effect size is calculated based on the number of available participants and/or based on the funding limitations. The wide range and a degree of subjectivity on clinical importance allows for the possibility of the sample size calculation being based on unrealistic expectations, which can exacerbate the problem. Indeed, a lack of consensus regarding clinically meaningful effect sizes within medical specialities has been reported (20).

The aim of this study was to examine if the assumed effects are greater than the observed effect estimates as a proxy for overoptimistic treatment effect assumptions at the trial design stage. The hypothesis was that sample size manipulation by selecting overoptimistic effect size at the design stage and vague or absent sample size calculations may contribute to research waste. Eighty-five SRs were deemed eligible from which 347 primary studies were identified. Within this cohort, the sample size was described in only 31.7% (110/347) of studies. From these 110 studies, in only 37 was the assumed clinical difference that the study was designed to detect reported (37/110). The assumed treatment effect was recalculated for the remaining 73 studies (73/110). We found that the assumed effect size was significantly larger than the observed effect size implying the possibility of overoptimistic assumptions.

Optimistic assumptions, flexibility in study design and statistical analyses with small, essentially underpowered studies are not immune to false positive (significant) results (2, 21). Small sample sizes are justified if it is established that the true effects being estimated are large enough to be reliably observed in such samples (21). Small studies can give statistically significant results only when they detect large effects, often by chance, a phenomenon known as the ‘winner’s curse’. Importantly, if the result in this ‘winning’ study is used to estimate the sample size that is required in duplicate studies, this will lead to a further perpetuation of overoptimistic assumed effects. Ultimately, both effect inflation and publication bias are likely to be worse in smaller versus larger studies. Hence, the confidence in the evidence for a large effect in small studies is diminished (21).

At the primary study level, the sample size was described in only 31.7% of studies. This may be an underestimation of the wider issue as only a single electronic database was searched in the current study. However, despite this, the findings reinforce concerns about the poor reporting and low reproducibility of sample size calculations within the orthodontic literature as a whole (8, 13). The inclusion of a methodologist at the design stage would be beneficial to ensure that adequate consideration is given to statistical analyses and clearly defining the sample size calculation prior to trial commencement. Increasing the sample size of primary inconclusive studies in consequent studies is challenging and may not necessarily confirm effectiveness (22). The overwhelming majority of primary orthodontic trials were single centre (78.1%). A large and well conducted single centre trial can still generate valid research findings but there is some evidence that the issues around small sample size can be improved through conducting multi-centre research (14). For the aforementioned reasons, this sort of trial will also likely have to undergo a more rigorous design process and will increase the pool of patients from which the trial can recruit. Prior registration and acceptance of studies by journals may help to encourage a more transparent process of hypothesis testing and reduce publication bias, ensuring negative findings are published and not confined to the grey literature (23). More accurate effect size assumptions can therefore be made to inform future research. It is advisable that assumptions about effect size from small pilot studies may not always be appropriate and alternative suggestions have included sample size reviews and the use of internal pilots within the design of a larger trial so that recalculation or adjustment to the sample size can be made, and clearly reported, during the progress of the trial (24).

## Conclusions

Only 31.7% of identified studies reported sample size calculations at the design stage. There was also a significant difference between assumed and observed treatment effects, suggesting greater values for the assumed effect sizes. Careful consideration of assumptions at the design stage is necessary to reduce the unreliability of clinical study results and research waste in orthodontic clinical research.

## Data Availability

The data underlying this article are available in the article and in its online supplementary material.
